# Lactate administration activates the ERK1/2, mTORC1, and AMPK pathways differentially according to skeletal muscle type in mouse

**DOI:** 10.14814/phy2.13800

**Published:** 2018-09-19

**Authors:** Hugo Cerda‐Kohler, Carlos Henríquez‐Olguín, Mariana Casas, Thomas E. Jensen, Paola Llanos, Enrique Jaimovich

**Affiliations:** ^1^ Faculty of Medicine Center for Exercise, Metabolism and Cancer ICBM Universidad de Chile Santiago Chile; ^2^ Laboratory of Exercise Science Clínica MEDS Santiago Chile; ^3^ Department of Nutrition, Exercise and Sports Molecular Physiology Group Faculty of Science University of Copenhagen Copenhagen Denmark; ^4^ Physiology and Biophysics Program ICBM Faculty of Medicine Universidad de Chile Santiago Chile; ^5^ Institute for Research in Dental Sciences Facultad de Odontología Universidad de Chile Santiago Chile

**Keywords:** metabolism, molecular signaling, skeletal muscle

## Abstract

Skeletal muscle is described as an endocrine organ, constitutively or intermittently secreting bioactive molecules. The signaling pathways by which these molecules mediate changes in skeletal muscle and regulate interorgan crosstalk are only partly understood. Lactate is widely described as a signaling molecule in different cells, but the role of lactate as a signaling molecule in mature skeletal muscle has not been fully unveiled. The aim of this study was to determine the role of lactate on activation of signaling pathways in adult mouse skeletal muscle. Male mice were injected intraperitoneally with lactate or saline, and tissues were dissected after 40 min. Phosphorylation levels of relevant proteins in muscle were assessed by Western blotting. After lactate administration, we found an increase in p‐ERK1/2^Thr202/Tyr204^ (3.5‐fold; *P* = 0.004) and p‐p70S6K^T^ ^hr389^ (1.9‐fold; *P* = 0.01) in quadriceps; and an increase in p‐rpS6^Ser235/236^ in both quadriceps (6.3‐fold; *P* = 0.01) and EDL (2.3‐fold; *P* = 0.01), without changes in soleus. There was a tendency toward an increase in p‐AMPK^T^ ^hr172^ (1.7‐fold; *P* = 0.08), with a significant increase in p‐ACC^S^ ^er79^ (1.5‐fold; *P* = 0.04) in soleus, without changes in quadriceps and EDL. These results support the hypothesis that lactate plays a role in the molecular signaling related to hypertrophy and to oxidative metabolism on adult skeletal muscle and suggest that this activation depends on the skeletal muscle type. The mechanisms that underlie the effect of lactate in mature skeletal muscles remain to be established.

## Introduction

Skeletal muscle represents ~40% of body weight and is integral for locomotion and metabolic health (Brook et al. 2016). Recent years have provided clear evidence for skeletal muscle being an endocrine organ, constitutively or intermittently secreting bioactive molecules (Weigert et al. 2014). The biologically active molecules released from skeletal muscle cells during muscle contraction exert autocrine, paracrine, and endocrine effects (Pedersen 2011; Pillon et al. 2012). The discovery of how these molecules mediate changes in skeletal muscle and regulate interorgan crosstalk could reveal novel therapeutic targets and support the development of personalized exercise training regimens.

Historically, lactate has often been incorrectly viewed as a dead‐end, fatigue‐causing waste product (Ferguson et al. 2018). Currently, the cell‐to‐cell lactate shuttle supports a role of lactate as an oxidative and gluconeogenic substrates (Brooks 2018). In skeletal muscle, lactate is converted to pyruvate in the cytosol, and is then used as a source of carbon for the tricarboxylic acid cycle (Hui et al. 2017). Recently, lactate has been described as a signaling molecule in adipocytes (Liu et al. 2009), astrocytes (Yang et al. 2014), liver (Lezi et al. 2013), and capillaries (Morland et al. 2017). In vivo lactate administration increases messenger RNA levels of oxidative metabolism‐related genes as PGC‐1*α* in skeletal muscle of mouse (Kitaoka et al. 2016). In skeletal muscle cell lines as L6 and C2C12, lactate activates the ERK1/2 pathway (Li et al. 2014) and is also related to both oxidative metabolism (Hashimoto et al. 2007; Kim et al. 2017), and hypertrophy signaling (Ohno et al. 2018; Oishi et al. 2015). However, the molecular signaling activated by lactate in mature skeletal muscle has not been fully unveiled.

We hypothesized that lactate plays a role in the molecular signaling related to hypertrophy and to oxidative metabolism in adult skeletal muscle. To test this hypothesis, we exposed mice to in vivo lactate administration and analyzed intracellular signaling activation in adult skeletal muscle.

## Methods

### Ethical approval

All experiments as well as the breeding protocol performed in Denmark were approved by the Danish Animal Experimental Inspectorate and complied with the European Convention for the Protection of Vertebrate Animals used for Experiments and other Scientific Purposes (authorization 2015‐15‐0201‐00477). The Bioethics Committee of the Faculty of Medicine, Universidad de Chile, approved all animal procedures performed in Chile (authorization CBA‐0822‐FMUCH). The experiments to assess phosphorylation of proteins were performed in Denmark, and the experiments to assess insulin and glucose levels were performed in Chile.

### Animals

Male mice were maintained on a 12:12 light‐dark cycle with unlimited access to standard rodent chow and water. Animals (C57BL/6JRj) for the experiment performed in Denmark were obtained from Janvier Labs (Le Genest St. Isle, France). The animals (C57BL/6J) for the experiment performed in Chile were obtained from the Animal Facility at the Faculty of Medicine (Universidad de Chile).

### Lactate administration

All mice were 10–12 weeks old at the start of the experiments. On the day of the experiment, mice were transferred to individual cages and were fasted for 5–6 h. Mice were anesthetized (6 mg pentobarbital sodium and 0.6 mg lidocaine /100 g body weight (Jensen et al. 2015)) and randomly assigned to either vehicle group (VEH) or lactate group (LAC). LAC mice received an intraperitoneal injection (IP) of sodium L‐lactate (≥99.0%, Aldrich, 71718; 3 g/kg body weight; dissolved in phosphate buffered saline; pH‐adjusted to 7.4). VEH mice received the same volume (per kg bodyweight) of phosphate‐buffered saline (sodium phosphate monobasic (Aldrich, S3139; 1.7 mmol/L); sodium phosphate dibasic (Merck, 567550; 8.1 mmol/L); sodium chloride (Aldrich, S5886; 147 mmol/L); pH 7.4). The dose of lactate was chosen from previous experiments to obtain values of blood lactate about 20 mmol/L, which are similar to those found after maximal exercise (Juel et al. 1990). Gross estimation of blood sodium and effective osmolality values after the sodium lactate injection suggest that values were within a normal range of variation in mice (Bekkevold et al. 2013; Otto et al. 2016).

### Blood lactate, glucose, and insulin measurements

Blood samples were obtained from the tail before and after lactate or vehicle administration. Basal blood lactate, glucose, and insulin levels (0 min) were measured before the IP injection. Lactate levels were measured 5, 15, and 30 min after the injection using a portable blood lactate analyzer (Lactate Plus, Nova Biomedical). Glucose levels were measured 15 and 30 min after the injection using a portable blood glucose analyzer (FreeStyle Optium, Abbott Diabetes Care). Submandibular bleeding method (Golde et al. 2005) was used for blood collection and to determine serum insulin concentrations at 15 and 30 min after the injection using a commercially available immunoassay specific for mice (Millipore). The quadriceps (Quad), *extensor digitorum longus* (EDL), and soleus (Sol) muscles were dissected at 40 min after lactate administration, immediately snap‐frozen, and stored at −80°C. The interval between snap‐freezing and assaying for phosphorylation of proteins was 1–2 days.

### Muscle immunoblotting

For evaluation of total and phosphorylated levels of proteins, lysates were prepared as previously described (Jensen et al. 2015). Muscles from VEH (*n* = 5) and LAC (*n* = 7) were homogenized in 300 *μ*L ice‐cold lysis buffer (50 mmol/L Tris·HCl, 150 mmol/L NaCl, 1 mmol/L EDTA, 1 mmol/L EGTA, 50 mmol/L NaF, 5 mmol/L Na4P2O7, 2 mmol/L Na3VO4, 1 mmol/L dithiothreitol, 1 mmol/L benzamidine, 1% Nonidet P‐40, and 0.5% protease inhibitor cocktail, pH 7.4) on a beadmill (Tissuelyzer II, Qiagen; 1 min, 30 Hz, maximum 10 muscles/round). Then, samples were rotated end‐over‐end for 30 min at 4°C and spun at 13,000*g* for 20 min to generate lysates. Protein determination (1:10 dilution) was performed by Bicinchoninic acid method (Pierce, Thermo Scientific) and lysates were separated on 10% SDS‐PAGE gels. After electrophoresis, proteins were transferred to polyvinylidene fluoride membranes (Bio‐Rad Laboratories). The primary antibodies used were phospho‐S6 Ribosomal Protein Ser235/236 (Cell Signaling Technology (CST), USA, cat#4858), phospho‐Erk1/2 Thr202/Tyr204 (CST, cat#9101), phospho‐AMPK*α* Thr172 (CST, cat#2535), phospho‐ACC Ser79 (CST, cat#11818), AMPK*α* Total (CST, cat#5831), phospho‐Akt Thr308 (CST, cat#4056), phospho‐Akt Ser473 (CST, cat#9271), Akt2 Total (CST, cat#9272), phospho‐TBC1D1 Ser237 (Millipore, USA, cat#07‐2268), phospho‐TBC1D4 Thr642 (CST, cat#8881), and phospho‐p70S6K Thr389 (CST, cat#9234). PDH‐E1*α* phosphorylation at Ser293 and Ser300 were determined using antibodies as previously described (Pilegaard et al. 2006). All blots were developed on a Chemidoc MP imaging system (Bio‐Rad Laboratories) using enhanced chemiluminescence (ECL^+^, Amersham Biosciences). To confirm equal loading of the samples, the blots were submerged in Coomassie brilliant G‐250 solution for 1 min (Moritz 2017), washed in distilled water and then left in de‐staining solution for 10–15 min before taking a picture using the Chemidoc MP imaging system. Signals were quantified (Image Lab version 4.0, BioRad Laboratories) and expressed as arbitrary units.

### Statistical analysis

All values were expressed as median with interquartile range (IQR). Nonparametric data were analyzed using two‐tailed Wilcoxon rank‐sum test (also known as Mann–Whitney). Statistical significance was set at *P* < 0.05. As a complementary analysis, we assessed the magnitude of the effect of the intervention via estimation of the nonparametric effect size (ES; with 95% confidence interval) (Ialongo 2016) according to formulas previously described (Ivarsson et al. 2013). The following threshold values for ES reported as *d* were employed: <0.5 as small, 0.5–0.79 as medium, 0.8–1.29 as large, and ≥1.3 as very large (Ialongo 2016). Statistical analysis was performed using STATA 13.0 (StataCorp, College Station, TX).

## Results

### Acute in vivo lactate administration increases blood lactate levels

**Figure 1:**
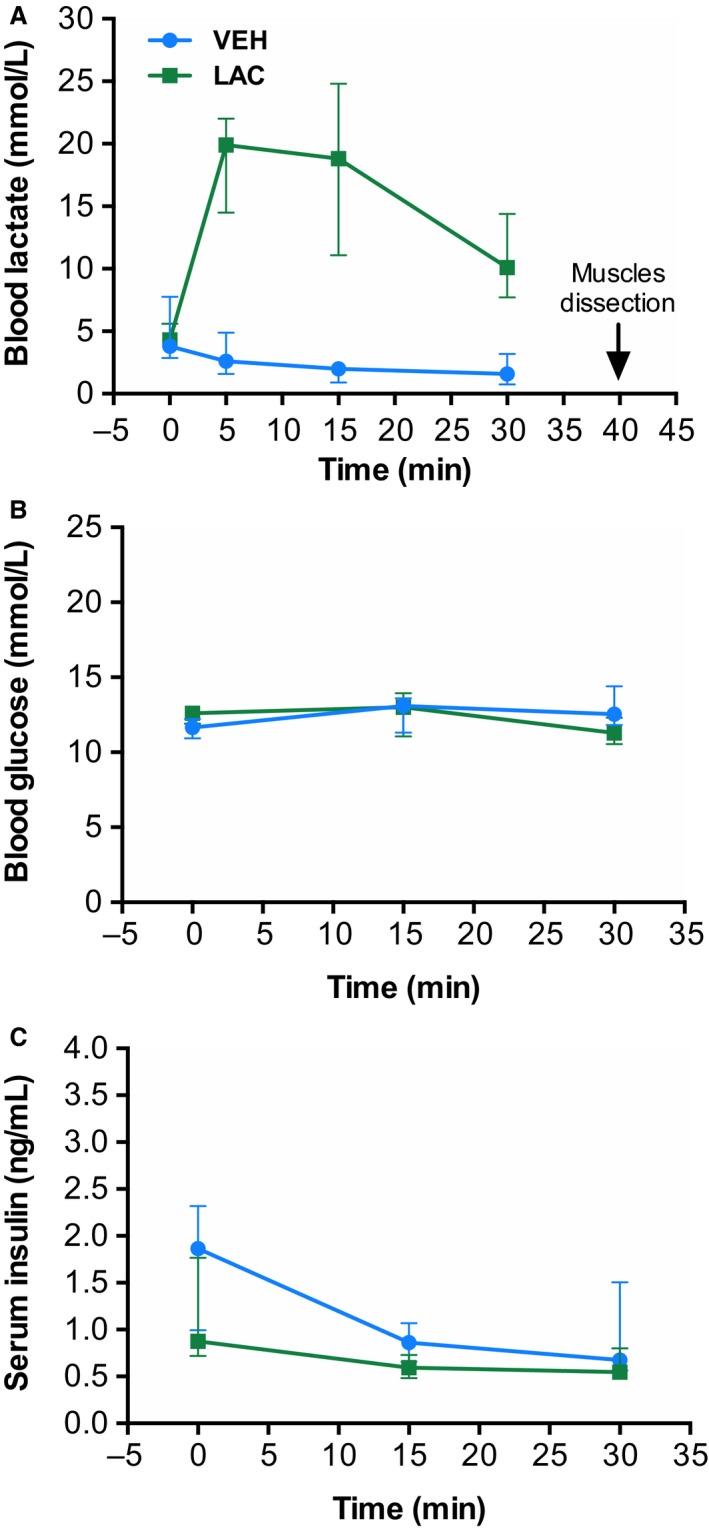
Lactate administration increases blood lactate levels without changes in blood glucose and insulin levels. Blood lactate (A), glucose (B), and insulin (C) levels after intraperitoneal injection of vehicle (VEH,* n* = 3–5, gray line) or lactate (LAC,* n* = 5–7, black line). The data are presented as the median and interquartile range.

To study the effect of in vivo lactate stimulation, anesthetized mice were lactate‐ or saline IP injected and tissues were harvested 40 min post‐injection. The time‐course of blood lactate after injection of lactate or vehicle is shown in Figure 1A. Blood lactate levels increased quickly after a single dose of lactate, but not after injection of the same volume of phosphate buffered saline. Lactate concentration peak was found between 5 and 15 min after the injection.

### Acute in vivo lactate administration increases blood lactate levels without changes in neither blood glucose nor insulin levels

Lactate is a gluconeogenic precursor in liver (Hui et al. 2017; van Hall 2010) and stimulates insulin secretion in beta cell lines (Meats et al. 1989). To explore if lactate induced an increase in blood glucose and insulin levels, we measured glucose and insulin during in vivo lactate administration. As represented in Figure 1, there were no changes in blood glucose (Fig. 1B) or in insulin levels (Fig. 1B) at any time in LAC group compared with the VEH group.

### Acute in vivo lactate administration induces intracellular signaling in adult skeletal muscle

**Figure 2:**
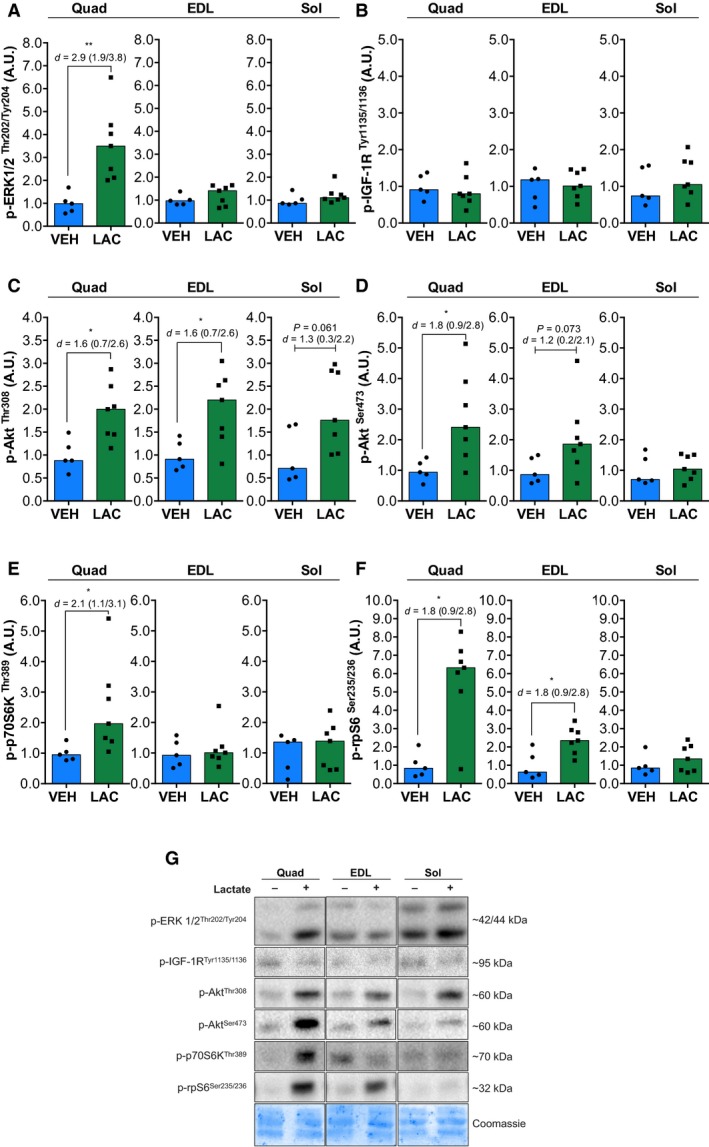
Lactate administration increases intracellular signaling related to ERK1/2 and Akt/mTORC1 pathways. Phosphorylation of ERK1/2^Thr202/Tyr204^ (A), IGF‐1R^T^ ^yr1135/1136^ (B), Akt^Thr308^ (C), Akt^Ser473^ (D), p70S6K^T^ ^hr389^ (E), and rpS6^Ser235/236^ (F) in quadriceps (Quad), *extensor digitorum longus* (EDL), and soleus (Sol) skeletal muscles after vehicle (VEH,* n* = 5) or lactate (LAC,* n* = 7) administration. Bars represent the median in the scatterplot. Representative immunoblots are shown in G. VEH, vehicle; LAC, lactate. Values in graphs are arbitrary units (A.U.). Statistical significance compared with vehicle is indicated by *P < 0.05 and **P < 0.01. *d*: effect size with 95% confidence interval.

Lactate activates MAPK pathways in skeletal myotubes (Li et al. 2014). To test whether lactate activates the ERK1/2 pathway in mature skeletal muscle, we measured ERK1/2^Thr202/Tyr204^ phosphorylation 40 min after in vivo lactate administration. As is shown in Fig. 2A, ERK1/2^Thr202/Tyr204^ phosphorylation was increased by lactate in Quad (3.5‐fold; *P *=* *0.004), without changes in EDL and Sol.

Lactate is correlated with myotubes hypertrophy and resistance training hypertrophy (Kawada and Ishii 2005; Ohno et al. 2018; Oishi et al. 2015). To explore the effect of lactate on anabolic pathways, we determined the activation of the canonical IGF‐1/Akt/mTORC1 pathway. No changes were observed in p‐IGF‐1R^Tyr1135/1136^ (Fig. 2B). Figure 2C shows that lactate administration induced an increase in phosphorylated Akt^Thr308^ in Quad (2.0‐fold; *P *=* *0.02) and EDL (2.2‐fold; *P *=* *0.02). There was a nonsignificant tendency toward an increase in Sol (1.7‐fold; *P *=* *0.06). Phosphorylation of Akt^Ser473^ (Fig. 2D) increased only in Quad (2.4‐fold; *P *=* *0.01). EDL muscle showed a nonsignificant tendency toward an increase (1.8‐fold; *P *=* *0.07).

**Figure 3:**
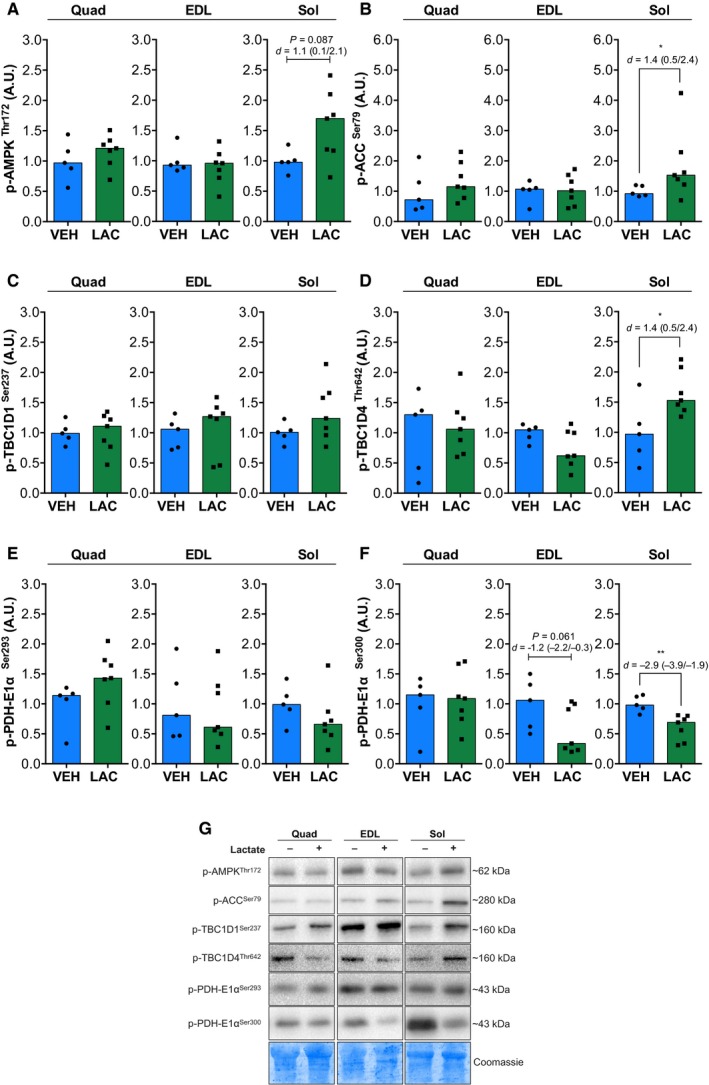
Lactate administration increases intracellular signaling related to AMPK pathway. Phosphorylation of AMPK^T^ ^hr172^ (A), ACC^S^ ^er79^ (B), TBC1D1^Ser237^ (C), TBC1D4^Thr642^ (D), PDH‐E1*α* ^Ser293^ (E), and PDH‐E1*α* ^Ser300^ (F) in quadriceps (Quad), *extensor digitorum longus* (EDL), and soleus (Sol) skeletal muscles after vehicle (VEH,* n* = 5) or lactate (LAC,* n* = 7) administration. Bars represent the median in the scatterplot. Representative immunoblots are shown in G. VEH, vehicle; LAC, lactate. Values in graphs are arbitrary units (A.U.). Statistical significance compared with vehicle is indicated by *P < 0.05 and **P < 0.01. *d*: effect size with 95% confidence interval.

We next investigated commonly used markers of mTORC1 signaling including p70S6K^Thr389^ and rpS6^Ser235/236^ (Ogasawara et al. 2016). Fig 3E shows that phosphorylation of p70S6K^Thr389^ was increased by lactate administration only in Quad (1.9‐fold; *P *=* *0.01). Lactate administration increased p‐rpS6^Ser235/236^ (Fig. 2F) in Quad (6.3‐fold; *P *=* *0.01) and EDL (2.3‐fold; *P *=* *0.01). There were no changes in Sol.

AMPK controls the expression of oxidative metabolism‐related genes and also regulates metabolism during exercise (Fritzen et al. 2015; Kjøbsted et al. 2018; McGee and Hargreaves 2010). We tested whether in vivo lactate administration activates the AMPK pathway. Figure 3A shows that lactate injection induced a nonsignificant increase in p‐AMPK^Thr172^ in Sol (1.7‐fold; *P *=* *0.08), without changes in Quad and EDL. Phosphorylation of ACC^Ser79^ only increased in Sol (1.5‐fold; *P *=* *0.04; Fig. 3B). There were no changes in phosphorylation of TBC1D1^Ser237^ in any muscle (Fig. 3C). Intriguingly, we only found an increase in phosphorylation of TBC1D4^Thr642^ in Sol (1.5‐fold; *P *=* *0.04, Fig. 3D), without changes in Quad and EDL.

AMPK regulates PDH activity and fuel selection in muscle metabolism (Fritzen et al. 2015). Therefore, we assessed PDH phosphorylation on the E1*α* subunit as a marker of its activity (Klein et al. 2007). Phosphorylation of PDH‐E1*α* ^Ser300^ decreased only in Sol (0.6‐fold; *P *=* *0.004; Fig.3F), but not in Quad and EDL muscles. Moreover, phosphorylation of PDH‐E1*α* ^Ser293^ (Fig. 3E) showed a nonsignificant tendency toward a decrease in Sol (0.6‐fold; *P *=* *0.1) and a nonsignificant increase in Quad (1.4‐fold; *P *=* *0.1).

## Discussion

This study showed for the first time that lactate in vivo administration activates intracellular signaling pathways in mature skeletal muscle. Our main finding was that in vivo lactate administration increased protein phosphorylation related to the ERK1/2, Akt/mTORC1, and AMPK pathways differentially depending on the skeletal muscle type.

**Figure 4:**
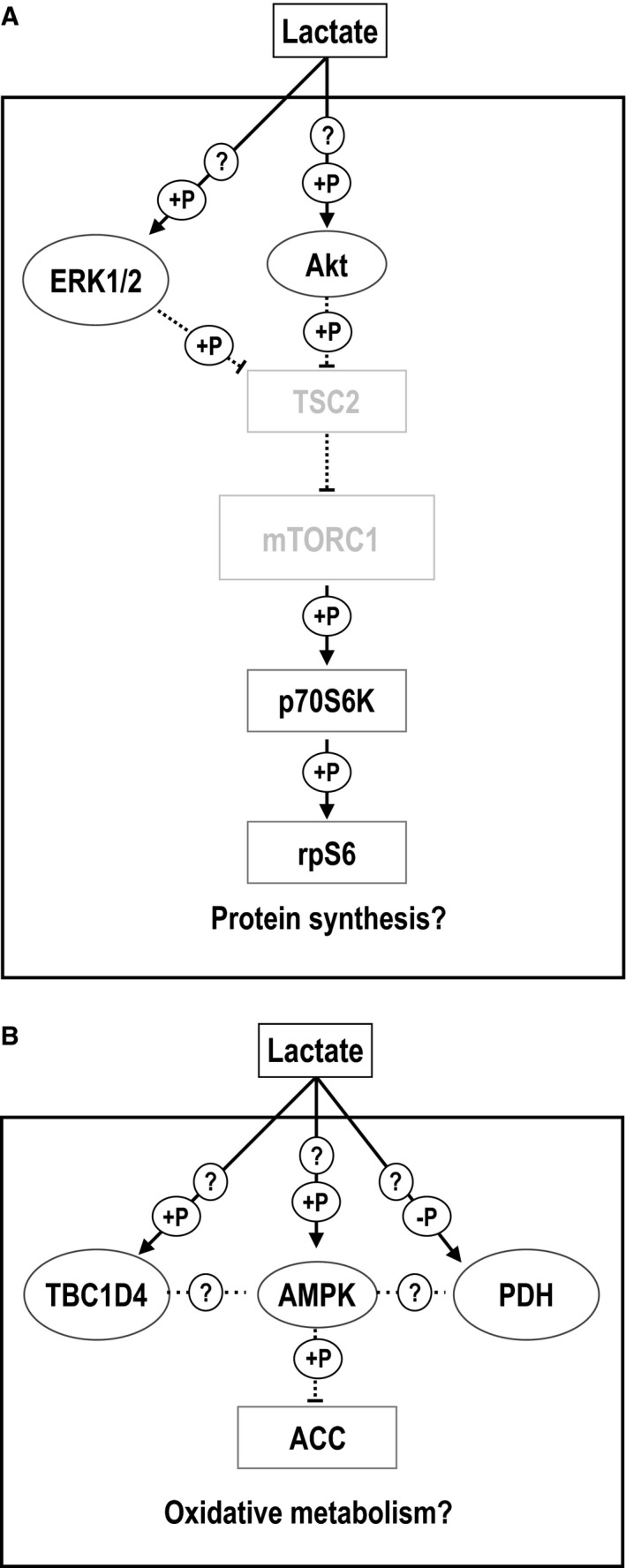
Lactate‐induced hypertrophic and oxidative signaling pathways activation in skeletal muscle. The working hypothesis for signaling pathways activated by in vivo lactate administration in mixed/fast (A) and slow (B) skeletal muscles. ERK1/2: extracellular signal‐regulated protein kinases 1 and 2; Akt: protein kinase B; TSC2: tuberous sclerosis complex 2; mTORC1: mammalian target of rapamycin complex 1; p70S6K: ribosomal S6 kinase; rpS6: ribosomal protein S6; AMPK: 5’‐AMP‐activated protein kinase; TBC1D4: TBC1 domain family member 4; ACC: Acetyl‐CoA carboxylase; PDH: pyruvate dehydrogenase. → represents activation; ···I represents inhibition. +P: increased phosphorylation; ‐P: decreased phosphorylation. ? represents unknown mechanisms.

Recent studies have shown that lactate activates the ERK1/2 pathway and regulates myogenesis (Willkomm et al. 2014), stimulates the anabolic signals regulating hypertrophy (Oishi et al. 2015), and increases myotube diameter (Ohno et al. 2018) in skeletal muscle cell lines. Here, we demonstrated that phosphorylation of ERK1/2^Thr202/Tyr204^ and p70S6K^Thr389^ was significantly increased in Quad, while phosphorylation of Akt^Thr308^, Akt^Ser473^, and rpS6^Ser235/236^ was significantly increased in both Quad and EDL, without significant changes in Sol muscle. These results are consistent with previous studies in C2C12 myotubes that show an increase in p‐ERK1/2^Thr202/Tyr204^ after incubation of 20 mmol/L lactate for 2 h (Ohno et al. 2018), and an increase in p‐p70S6K^Thr389^ after incubation of 10 mmol/L lactate for 6 h (Oishi et al. 2015). Taken together, these findings showed that in vivo lactate administration is sufficient to activate proteins related to ERK1/2 and Akt/mTORC1 pathways. The results suggest that lactate is involved in the signaling network related with protein synthesis in skeletal muscle, preferentially (if not exclusively) in mixed/glycolytic adult skeletal muscles type as Quad and EDL (Fig. 4A), but not in oxidative muscles as Sol.

Lactate increases mRNA levels of genes related to oxidative metabolism in L6 myotubes (Hashimoto et al. 2007) and mature skeletal muscle (Kitaoka et al. 2016). AMPK activation enhances muscle fiber oxidative metabolism by stimulating mitochondrial biogenesis (Kjøbsted et al. 2018). Interestingly, Hoshino and colleagues (Hoshino et al. 2015) showed that prior administration of dichloroacetate decreased lactate accumulation in blood and muscle during exercise which tended to decrease phosphorylation of AMPK^Thr172^, suggesting a role of lactate in AMPK activation in skeletal muscle. In this study, we found that lactate stimulation trended toward increasing the phosphorylation of AMPK^Thr172^ and significantly increased ACC^Ser79^ in Sol, without changes in Quad and EDL. Interestingly, in soleus we found an increase in the phosphorylation of TBC1D4^Thr642^, without changes in p‐TBC1D1^Ser237^. Since p‐TBD1D4^Thr642^ is not a substrate of AMPK (Kjøbsted et al. 2018), these results suggest an alternative mechanism for regulation of this protein. Recently, Fritzen and colleagues demonstrated that AMPK*α*2 regulates muscle metabolism through inhibition of the PDH complex (Fritzen et al. 2015). Here, we observed a significant decrease in p‐PDH‐E1*α* ^Ser300^ in Sol, a tendency toward a decrease in EDL, without changes in Quad, suggesting that indeed this pathway could be regulated this way. We found no changes in phosphorylation of PDH‐E1*α* ^Ser293^ in any muscle. Collectively, these results showed that in vivo lactate administration activated AMPK and downstream targets mainly in oxidative skeletal muscle type as Sol, supporting the hypothesis that lactate may be involved in the signaling network related with the oxidative metabolism (Fig. 4B).

In summary, our results support the hypothesis that lactate plays a role in the activation of signaling pathways related to hypertrophy in mixed/fast muscles via Akt/mTORC1 and ERK1/2 and that it also plays a role in regulating oxidative metabolism via AMPK in slow adult skeletal muscle. Our results suggest that, due to the differential effect of lactate administration in different muscle types, lactate signaling could be reinforcing the muscle phenotype after exercise. Our study highlights the relevance of understanding how lactate mediates the molecular signaling on skeletal muscles, and we cannot rule out the existence of interorgan crosstalk. The mechanisms that underlie the effect of lactate in mature skeletal muscles remain to be established.

## Conflict of Interests

The authors declare there are no conflicts of interest.
